# Supplementary material for: Evaluation of Spectral Imaging for Early Esophageal Cancer Detection
Source: Cancers (Basel). 2025 Jun 19;17(12):2049. doi: 10.3390/cancers17122049 (PMC12190529; doi:10.3390/cancers17122049)
Supplement: Supplementary file 1 [file cancers-17-02049-s001.zip › cancers-3653756-supplementary.pdf]

cancers

Article

Evaluation of spectral imaging for early esophageal cancer detection: Supplementary Material

Li-Jen Chang [1], Chu-Kuang Chou [1,2,3], Arvind Mukundan [4], Riya Karmakar [4], Tsung-Hsien Chen [5], Syna Syna [6], Chou-Yuan Ko [7,*], and Hsiang-Chen Wang [4,8,9,*]

1 Division of Gastroenterology and Hepatology, Department of Internal Medicine, Ditmanson Medical Foundation Chia-Yi Christian Hospital, Chia-Yi 60002, Taiwan; cych07235@gmail.com (L.-J.C.); vacinu@gmail.com (C.-K.C.)
2 Obesity Center, Ditmanson Medical Foundation Chia-Yi Christian Hospital, Chia-Yi 60002, Taiwan
3 Department of Medical Quality, Ditmanson Medical Foundation Chia-Yi Christian Hospital, Chiayi 60002, Taiwan
4 Department of Mechanical Engineering, National Chung Cheng University, 168, University Rd., Min Hsiung, Chia Yi 62102, Taiwan; karmakarriya345@gmail.com (R.K.); d09420003@ccu.edu.tw (A.M.)
5 Department of Internal Medicine, Ditmanson Medical Foundation Chia-Yi Christian Hospital, Chiayi 60002, Taiwan; cych13794@gmail.com (T.-H.C.)
6 Chitkara University, Department of Computer Science and Engineering, Chandigarh-Patiala National Highway NH- 64 Village Jansla, Rajpura, Punjab 140401, India; synab6498@gmail.com (S.S.)
7 Department of Gastroenterology, Kaohsiung Armed Forces General Hospital, 2, Zhongzheng 1st. Rd., Lingya District, Kaohsiung City 80284, Taiwan; Gastroenterokjy@gmail.com (C.-Y.K.)
8 Department of Medical Research, Dalin Tzu Chi Hospital, Buddhist Tzu Chi Medical Foundation, No. 2, Minsheng Road, Dalin, Chiayi, 62247 Taiwan.
9 Director of Technology Development, Hitspectra Intelligent Technology Co., Ltd., Kaohsiung 80661, Taiwan
* Corresponding Author: Gastroenterokjy@gmail.com (C.-Y.K.); hcwang@ccu.edu.tw (H.-C.W.)

Simple Summary: The eighth most frequent disease worldwide and the sixth main cause of cancer death is esophageal cancer (EC). EC must be detected early to prevent patient decline. This study uses SAVE technology to hyperspectral convert the EC image for spectrum information. YOLOv9, YOLOv10, RT-DETR, Roboflow 3.0, and YOLO-NAS models will be used to assess EC spectrum data using deep learning. This creates a model for detecting malignant tumors' stage and location. When specificity and sensitivity were assessed across all models, SAVE outperformed WLI. In the assessment, SAVE improved precision and F1 scores for most models, which is significant in patient care and therapy. Although Roboflow 3.0 is more sensitive to SCCs, YOLO-NAS operates effectively in all cases. These results show how different machine learning models perform after applying the recommended SAVE imaging approach under clinically relevant conditions.

Abstract: Esophageal carcinoma (EC) is the eighth most prevalent malignancy globally and the sixth leading cause of cancer-related mortality. Early identification of EC is essential to prevent further deterioration of the patient's health. This work aims to obtain spectrum information using hyperspectral conversion of the EC image with spectrum-aided vision enhancer (SAVE) technology. Subsequently, the information pertaining to the EC spectrum will be evaluated using deep learning methodologies. Specifically, the models RT-DETR, Roboflow 3.0, YOLO-NAS, YOLOv9, and YOLOv10 will be employed. A detection model will be developed to ascertain the stage and location of malignant lesions. The analysis of specificity and sensitivity across all models revealed that the SAVE modality outperformed the WLI modality. For instance, the examination revealed that SAVE improved the precision and F1 scores of most models, a finding that is crucial in the realm of patient care and treatment. YOLO-NAS and Roboflow 3.0 exhibited superior

performance among all models, with Roboflow 3.0 demonstrating greater sensitivity to SCCs compared to YOLO-NAS, which excels across all scenarios. The results demonstrate the performance of various machine learning models following the implementation of the suggested SAVE imaging technology in therapeutically relevant scenarios.

Keywords: Esophageal cancer ; SAVE; machine learning; Artificial Intelligence; YOLO; Narrow-band Imaging; White-light Imaging.

1. Dataset

Table S1. Shows the dataset instances of the classes before and after augmentation

Before Augmentation	
0. A: Dysplasia	601
1. B: SCC	159
2. C: Bleeding	213
3. D: Inflammation	81
4. E: through	1292
5. I: Cardia	323
6. J: Object	643
7. K: Tool	51
8. S: Artificial information	164
9. T: Bubble	661
10. U: Reflection	1068
Total	2063
After Augmentation	
0. A: Dysplasia	1202
1. B: SCC	318
2. C: Bleeding	426
3. D: Inflammation	162
4. E: through	2584
5. I: Cardia	646
6. J: Object	1285
7. K: Tool	102
8. S: Artificial information	328
9. T: Bubble	1322
10. U: Reflection	2134
Total	4125

2. About Classes

In this study, 12 different categories have been grouped that are essential for thorough examination of the model. Specifically, 12 classes have been considered, to ensure that the model does not misinterpret the cancer .Dysplasia signifies the growth of cells in the esophagus a sign of potential cancer development. It is only the true precursor lesion of EC Squamous Cell Carcinoma(SCC) is a form of cancer originating from the squamous cells that line the esophagus. Polyps are growths of the tissues that can be either benign or malignant, often discovered during procedures. Bleeding indicates the esophageal conditions, including cancer suggesting possible ulcers or lesions. Inflammation in the esophagus triggered by irritation or infection can make individuals more prone to developing alterations. The class through refers to the infiltration of cancer cells through the layers of the wall playing a role, in determining how severe the cancer is staged. The cardia marks the area where the esophagus meets with the stomach often examined for signs of esophageal cancer spreading. Tools encompass instruments utilized in both examinations and surgical interventions. The object class refers to any objects detected in the esophagus that could disrupt the procedures. Artificial information refers to data that may be redundant, and hence affect the model's accuracy. Bubbles indicates the presence of air affecting interpretations of conditions affecting the esophagus .Reflect indicates to how esophageal tissue reflects light during imaging offering information, about the esophagus structure and health. These categories collectively improve the precision and detail of detecting and diagnosing cancer.

3. Evaluation Indices

Evaluation metrics are quantitative, mathematical, and objective measures for grading the accuracy, performance, or the efficacy of statistical or machine learning algorithms[1]. These yield crucial information pertaining to understanding the performance of the model and can help in the comparison of different models, returns or the very same model, with the same algorithm configurations**.** Accuracy measures the percentage of samples in the data set that were correctly categorized by the model as positive, thus measures the capacity of the model to avoid the false positives.

$$\text{Accuracy} = \frac{TP+TN}{TP+TN+FP+FN} \tag{S-1}$$

Recall computes the ratio of correct positive cases relative to all the cases that are positive actually; it deals with the capacity of the model to diagnose all instances of a particular class.

$$\begin{aligned} \text{Precision} &= \frac{tp}{tp+fp} \\ \text{Recall} &= \frac{tp}{tp+fn} \end{aligned} \tag{S-2}$$

The F1 Score, which is formed by the precision score and the recall score, and summing them. The F-1 Score, therefore, delivers a more accurate account of the performance of the model in the balanced mode either of false positives or false negatives.

$$F = 2 \cdot \frac{\text{precision} \cdot \text{recall}}{\text{precision} + \text{recall}} \tag{S-3}$$

Recall values range from 0 to 1 and the average precision (AP) means have been computed on average precision values. mAP formula is based on the following sub metrics: Confusion matrix,Intersection over Union (IoU), Miss rate ,Hit rate. Four characteristics are necessary for developing a confusion matrix which is True Positive (TP) ,True Negatives (TN), True Negative (TN) and False Negatives (FN) .mAP50 is interpreted as the Mean average precision which is calculated at intersection over union (IoU) of 0. 50. It relates to how well the model performs based on the subset of detections that every algorithm should ideally be able to handle easily .mAP50-95 is the mean average precision at

the intersection of union of different levels of IoU thresholds from 0.50 to 0.95. Thus, it provides an overview of the model's performance depending on how difficult it is to find an object in an image.

$$\text{mAP} = \frac{1}{N}\sum_{i=1}^{N} \text{AP}_i \tag{S-4}$$

4. SAVE

The discrete alteration formulas to adapt the 24-color patch image and 24 color patch reflectance spectrum data to XYZ color space are as follows

On the camera part: alter sRGB color gamut space to XYZ color gamut space

$$\begin{bmatrix} X \\ Y \\ Z \end{bmatrix} = [M_A][T]\begin{bmatrix} f(R_{sRGB}) \\ f(G_{sRGB}) \\ f(B_{sRGB}) \end{bmatrix} \times 100, 0 \le \begin{matrix} R_{sRGB} \\ G_{sRGB} \\ B_{sRGB} \end{matrix} \le 1 \tag{S-5}$$

$$[T] = \begin{bmatrix} 0.4104\ 0.3576\ 0.1805 \\ 0.2126\ 0.7152\ 0.0722 \\ 0.0193\ 0.1192\ 0.9505 \end{bmatrix} \tag{S-6}$$

$$f(n) = \begin{cases} (\frac{n+0.055}{1.055})^{2.4}, n > 0.04045 \\ \left(\frac{n}{12.92}\right), otherwise \end{cases} \tag{S-7}$$

$$[M_A] = \begin{bmatrix} X_{SW}/X_{CW} & 0 & 0 \\ 0 & Y_{SW}/Y_{CW} & 0 \\ 0 & 0 & Z_{SW}/Z_{CW} \end{bmatrix} \tag{S-8}$$

$$Z = k\int_{400nm}^{700nm} S(\lambda)R(\lambda)\bar{z}(\lambda)d\lambda \tag{S-9}$$

$$k = 100/\int_{400nm}^{700nm} S(\lambda)\bar{y}(\lambda)d\lambda \tag{S-10}$$

The nonlinear response of the camera can be modified by a third-order formula, and the nonlinear response alteration variable is demarcated as $V_{Non\text{-}linear}$.

$$V_{Non-linear} = [X^3\ Y^3\ Z^3\ X^2\ Y^2\ Y^2\ X\ Y\ Z\ 1]^T \tag{S-11}$$

Within the dark current component of an imaging device, the dark current typically maintains a consistent value regardless of the quantity of light received. As such, a fixed value is assigned to represent the dark current's contribution, and the dark current modification variable is established accordingly VDark.

$$V_{Dark} = [a] \tag{S-12}$$

The variable matrix V is gotten by standardizing the product of VColor and VNonlinear, with the addition of VDark. To prevent over-correction, the standardization is limited to the third order.

$$V_{Color} = [XYZ\ XY\ XZ\ YZ\ X\ Y\ Z]^T \tag{S-13}$$

$$V = \begin{bmatrix} X^3\ Y^3\ Z^3 \\ X^2Y\ X^2Z\ Y^2Z \\ XY^2\ XZ^2\ YZ^2 \\ XYZ\ X^2\ Y^2\ Y^2 \\ XY\ XZ\ YZ\ X\ Y\ Z\ a \end{bmatrix}^T \tag{S-14}$$

Prior to utilizing CIE DE2000 for color difference computation, it is necessary to convert $XYZ_{Correct}$ and $XYZ_{Spectrum}$ from the XYZ color space to the lab color space. The equation for conversion is as ensues:

$$\begin{gathered} L^* = 116f\left(\frac{Y}{Y_n}\right) - 16 \\ a^* = 500\left[f\left(\frac{X}{X_n}\right) - f\left(\frac{Y}{Y_n}\right)\right] \\ b^* = 200\left[f\left(\frac{Y}{Y_n}\right) - f\left(\frac{Z}{Z_n}\right)\right] \end{gathered} \tag{S-15}$$

$$f(n) = \begin{cases} n^{\frac{1}{3}}, n > 0.008856 \\ 7.787n + 0.137931, otherwise \end{cases} \tag{S-16}$$

$$X = k\int_{400nm}^{700nm} S(\lambda)R(\lambda)\bar{x}(\lambda)d\lambda, \quad \text{(S-17)}$$
$$Y = k\int_{400nm}^{700nm} S(\lambda)R(\lambda)\bar{y}(\lambda)d\lambda, \quad \text{(S-18)}$$
$$Z = k\int_{400nm}^{700nm} S(\lambda)R(\lambda)\bar{z}(\lambda)d\lambda, \quad \text{(S-19)}$$
$$k = 100/\int_{400nm}^{700nm} S(\lambda)\bar{y}(\lambda)d\lambda. \quad \text{(S-20)}$$

In summary, the SAVE conversion technique established in this research offers a means to convert standard WLI endoscopic images into representations akin to NBI, thereby improving the visibility of tissue structures and vascular patterns critical for cancer detection. A Macbeth color checker was employed to calibrate the images for accurate color conversion, juxtaposing them with precise color data acquired from a spectrometer. This calibration process rectified discrepancies in lighting and camera response, facilitating uniform and clinically dependable color representation. The algorithm converted color information into a standardized color space (CIE 1931 XYZ) to align with the spectral properties of NBI. PCA was utilized to efficiently compress and reconstruct the spectral data, ensuring that the resultant images retained the critical visual characteristics necessary for medical interpretation. Subsequent modifications addressed lighting conditions and minimized color discrepancies, validated through the assessment of color fidelity and image congruence utilizing standard metrics such as SSIM and PSNR.

Some limitations of this study are worth discussing. One major limitation is that the data were collected within a single hospital only. This limitation may bring in bias from the institutions that the data belong to and the patients whom the data were collected from. As a result, the validity of the findings is low, and the conclusions drawn in the study are not for general use. Data across multiple centers in different countries and with different ethnicities must be collected to strengthen the generalizability of the findings and the proposed model in future investigations [2]. Another limitation is derived from the preprocessing step of the original image sizes of different dimensions into a common size of 640 pixels on each side of the images. This preprocessing procedure may have neglected relevant image details that could have affected the models' performance, though being helpful in managing the computational resources. Thus, future works are suggested to preserve higher resolution during the preprocessing or use adaptive resolution strategies to retain vital data and reduce calculative time. Another issue relates to the computational requirements needed for HSI and ML algorithms. The highly involved calculations pose a challenge by consuming much time and other resources. Thus, future research works must further investigate the use of better algorithms and the application of high-end computing technologies like graphics processing unit (GPU) and tensor processing unit (TPU). The goal of achieving real-time detection, which could be within microseconds, requires improvement of the software and hardware components and the incorporation of specific accelerators meant for accelerating specific operations within the computer, such as GPUs, field-programmable gate arrays, application-specific integrated circuits, and TPUs [3]. By contrast, ensemble learning unites several models to enhance their accuracy while increasing the reliability of the system for clinical application [4]. These approaches can be added to the usability of the technology, making it considerably easier for healthcare givers, including endoscopists, and increasing the reliability of the results they provide. The approach conducted here in EC could easily be applied to other types of cancers and perhaps in other medical afflictions where imaging is the best bet in early detection. Future research should aim at looking at the ability of the system to handle several diseases so that it could provide more comprehensive information. The study could be carried out on other forms of cancer, such as gastrointestinal cancer and breast

cancer. Other forms of cancer could help in establishing the versatility of the system. Moreover, the creation of a system that could detect and diagnose cancer in real-time considerably changes the approach to cancer therapy. The prospects for these developments could significantly improve patients' quality of life by enabling early diagnosis and immediate condition management. Further developments in medical imaging techniques based on the data of the presented study and other similar investigations could play an important role in improving the diagnostic capabilities and assessing the outcomes of patient treatment. Strengthening the study to assess the applicability of the SAVE system in various settings increases its credibility and effectiveness determinations. The data from KMUH can be used to assess the efficiency of imaging methods and the possible connections between lifestyle variables and progression of EC. This assessment is a valuable addition to oncology knowledge in general. The current study can serve as a good starting point. However, additions and improvements are needed. Therefore, future studies should further enhance its limitations and look for new possibilities for using HSI and ML in medical diagnostics. Thus, advancing the technology to include the most basic technology, diversifying the population of the study, and exploring different diseases that this device may be applied to could help in creating a stable diagnostic tool.

5. Results

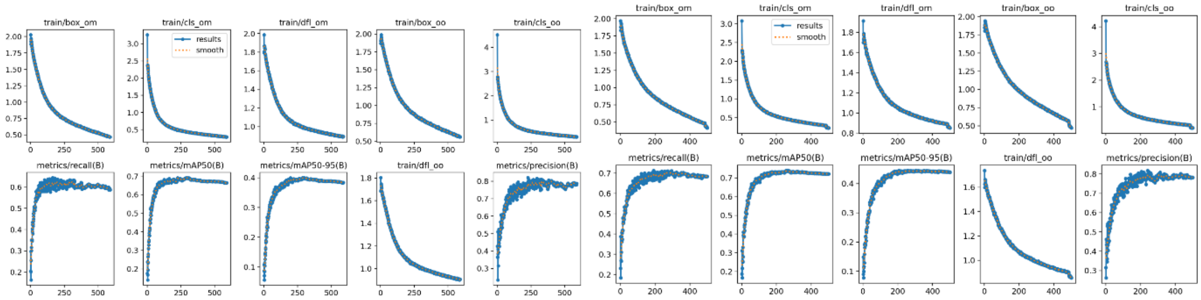

Figure S1. Visualization of training loss and performance metrics (precision, recall, and F1-score) for YOLOv10 models using both WLI and SAVE imaging modalities. The plots illustrate the learning curves during training and validation phases, highlighting differences in convergence behavior and overall model performance between the two imaging approaches.

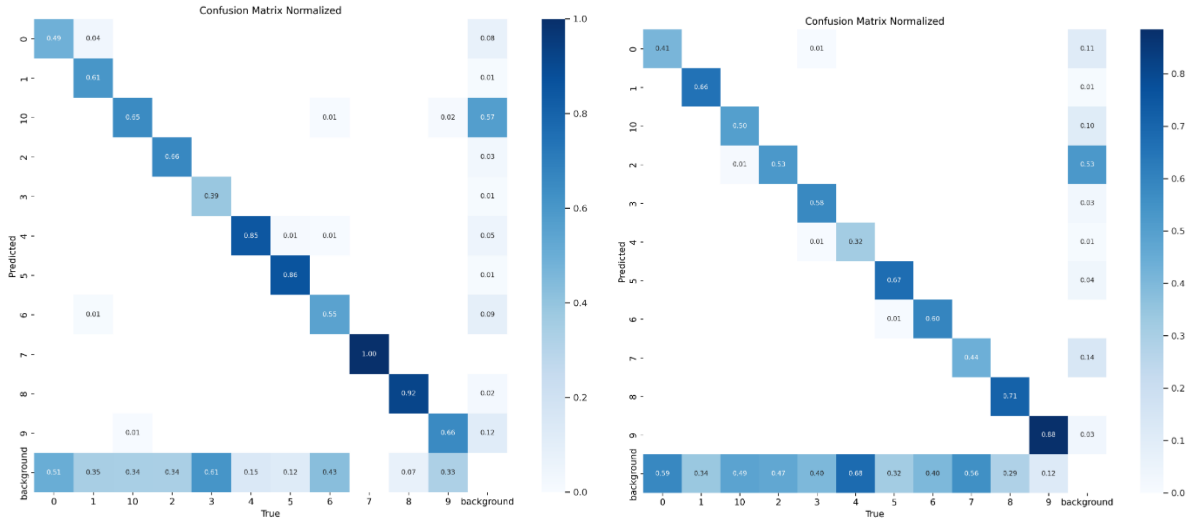

Figure S2 Confusion matrices for YOLOv10 model performance on WLI and SAVE imaging modalities. The matrices display the classification outcomes for each lesion class (normal, dysplasia, SCC, and inflammation), allowing comparison of true positives, false positives, and false negatives between WLI and SAVE.

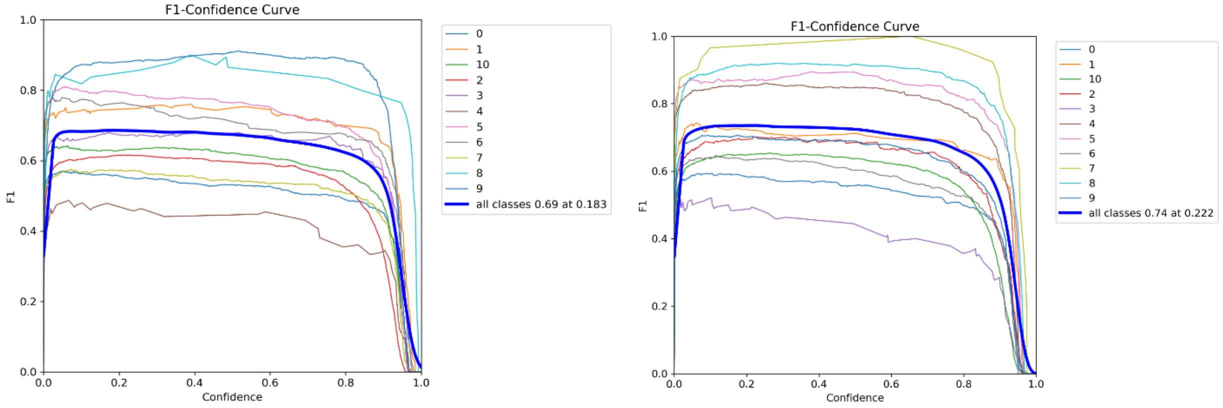

Figure S3 F1-score confidence curves for the YOLOv10 model using WLI and SAVE imaging modalities. The curves illustrate the relationship between model confidence thresholds and corresponding F1-scores, demonstrating the impact of varying confidence levels on classification performance for each imaging approach.

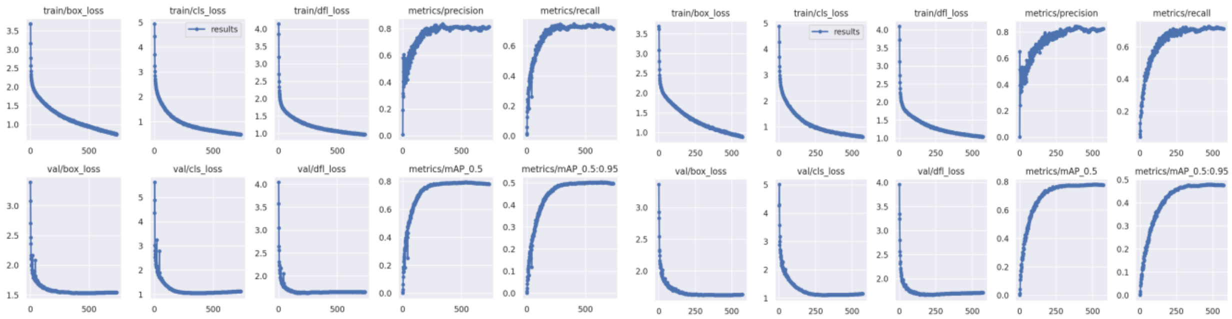

Figure S4. Visualization of training loss and performance metrics (precision, recall, and F1-score) for YOLOv9 models using both WLI and SAVE imaging modalities. The plots illustrate the learning curves during training and validation phases, highlighting differences in convergence behavior and overall model performance between the two imaging approaches.

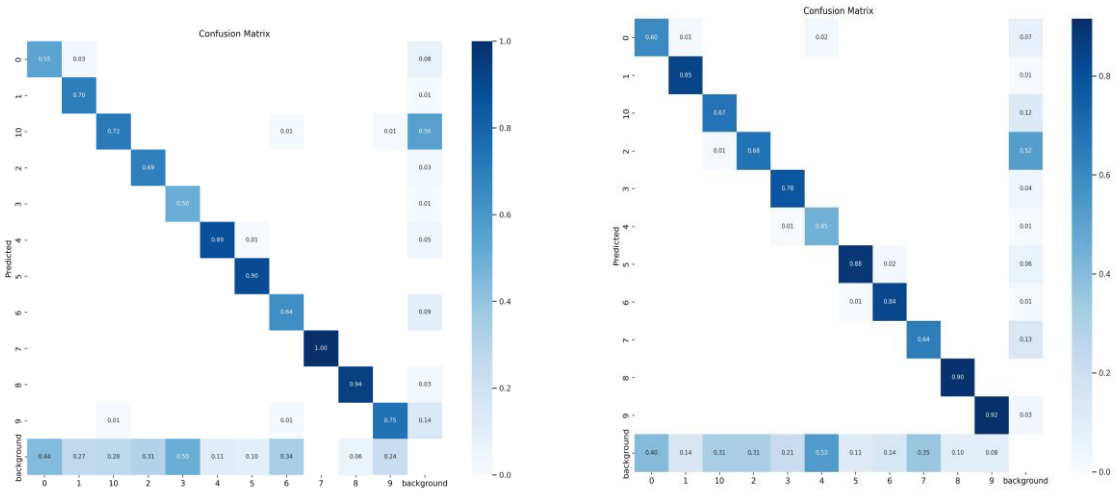

Figure S5 Confusion matrices for YOLOv9 model performance on WLI and SAVE imaging modalities. The matrices display the classification outcomes for each lesion class (normal, dysplasia, SCC, and inflammation), allowing comparison of true positives, false positives, and false negatives between WLI and SAVE.

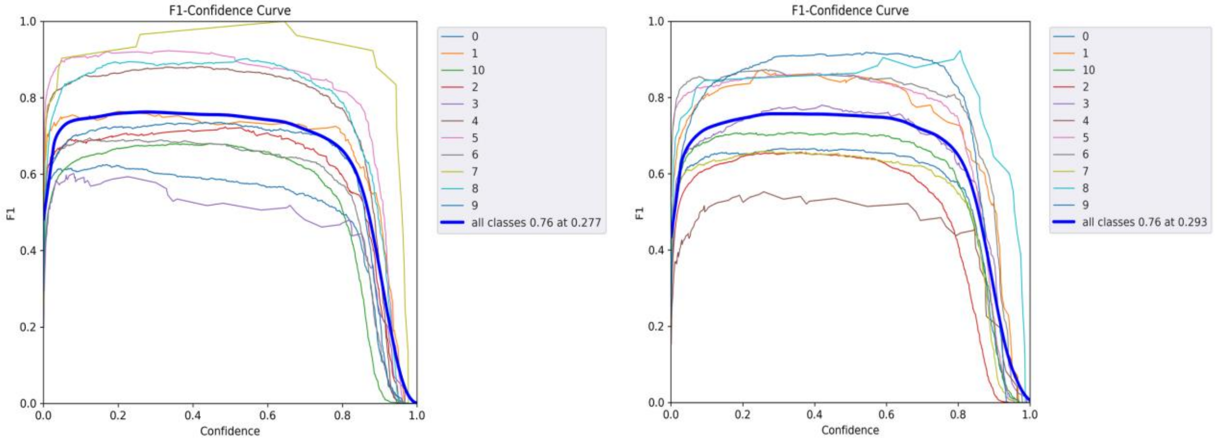

Figure S6 F1-score confidence curves for the YOLOv9 model using WLI and SAVE imaging modalities. The curves illustrate the relationship between model confidence thresholds and corresponding F1-scores, demonstrating the impact of varying confidence levels on classification performance for each imaging approach.

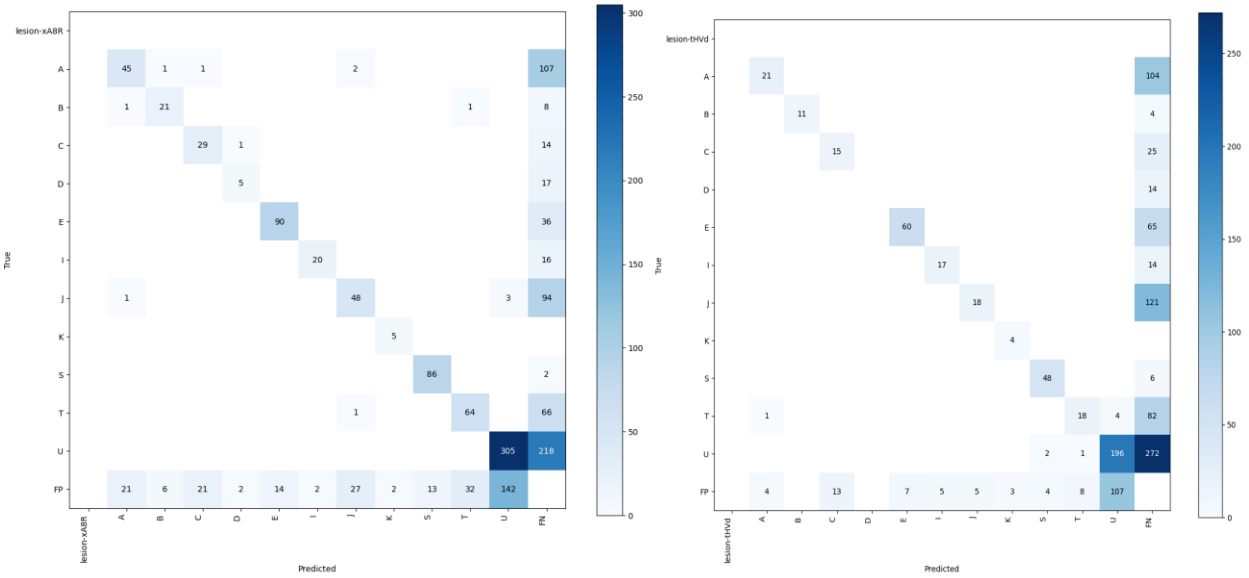

Figure S7 Confusion matrices for RT-DETR model performance on WLI and SAVE imaging modalities. The matrices display the classification outcomes for each lesion class (normal, dysplasia, SCC, and inflammation), allowing comparison of true positives, false positives, and false negatives between WLI and SAVE.

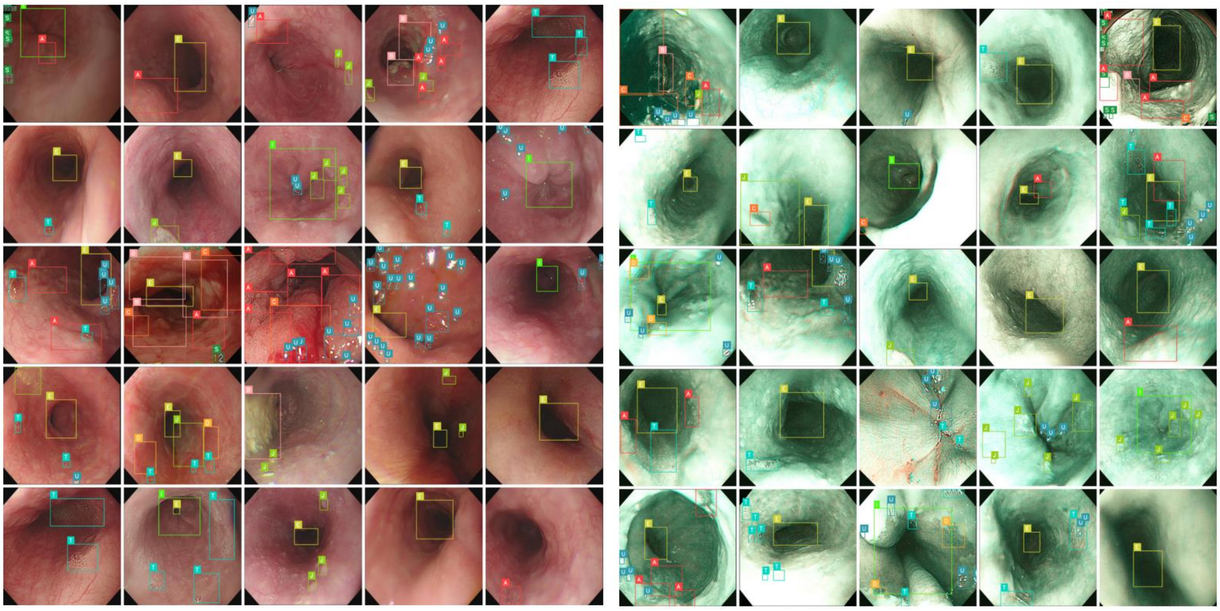

Figure S8. Predicted lesion detection results for WLI and SAVE images using the RT-DETR model. The figure showcases the bounding box predictions and class labels generated by the model, illustrating differences in detection performance between the two imaging modalities.

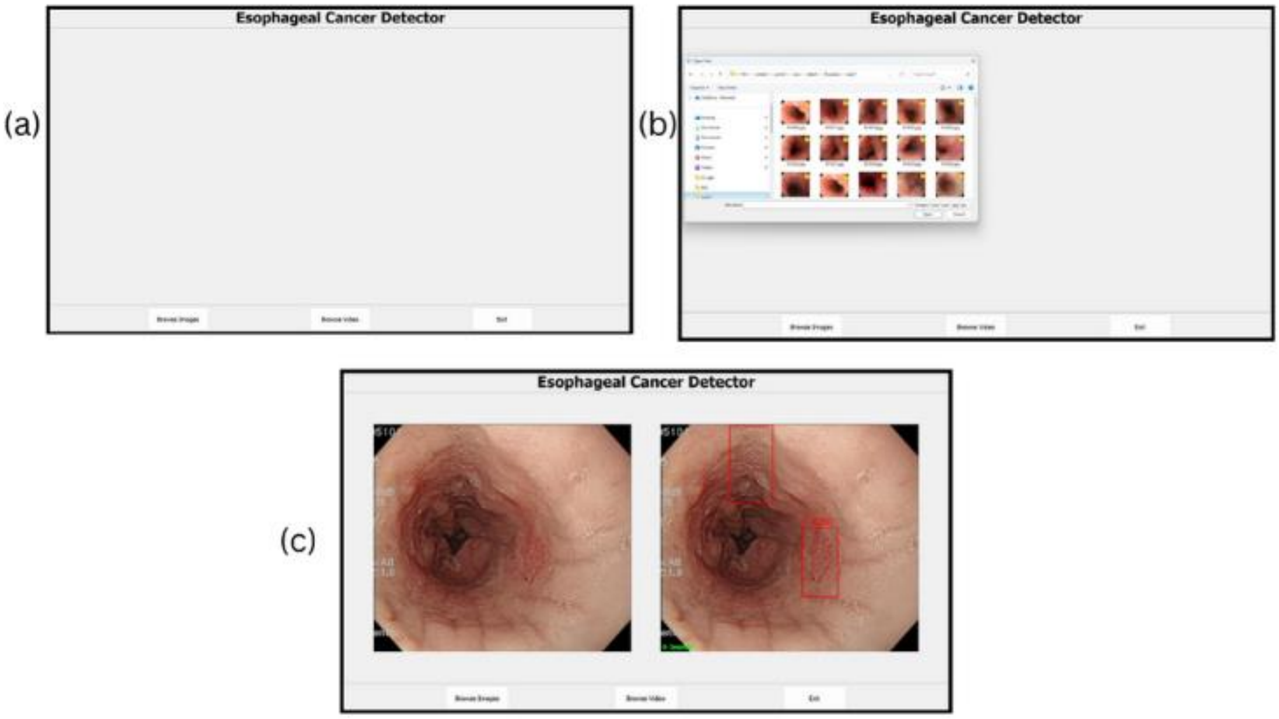

Figure S9. Screenshots of the windows based CAD application used for esophageal cancer detection

6. Machine learning models

6.1 YOLO models

The formula takes into account the predicted bounding boxes (pred_bboxes), the target bounding boxes (target_bboxes), and a foreground mask (fg_mask) for calculating the loU loss as follows:

$$\text{IoU} = \text{IoU}\left(\text{pred\_bboxes[fg\_mask]}, \text{target\_bboxes[fg\_mask]}\right), \quad (S21)$$

$$\text{IoU Loss} = \sum \left((1 - \text{IoU}) \cdot \text{weight}\right) / \text{target\_scores\_sum}. \quad (S22)$$

The final accumulation of loss for Compute Loss and ComputeLossLH. Both losses sum the losses from bbox, class, and DFL with the following weights.

$$\text{Total Loss} = (\text{box\_loss} \times 7.5) + (\text{cls\_loss} \times 0.5) + (\text{dfl loss} \times 1.5) \quad (S23)$$

The calculation formula for PPYoLOELoss/loss_iou is as follows:

$$\text{Loss}_{\text{iou}} = 1 - \frac{|B \cap B_{gt}|}{|B \cup B_{gt}|}, \quad (S24)$$

where the predicted bounding box is, and B_{gt} is the ground truth bounding box.

DFL enhances the accuracy of regressions corresponding to the bounding box coordinates by emphasizing the most relevant distribution bins for each coordinate. The formula to compute the PPYoloELoss/loss_df is as follows:

$$\text{Loss}_{df} = \frac{1}{N}\sum_{i=1}^{N} \sum_{j=1}^{4} \sum_{k=0}^{k-1} - y_{i,\text{j,k}} \log\left(\hat{y}_{i,j,k}\right), \quad (S25)$$

where N is the number of samples, j indexes the four coordinates of the bounding box, K is the number of discrete bins in the distribution, y(i, j, k) is the ground truth probability for bin k for coordinate j of sample i, and y ˆ(i, j, k) is the predicted probability. PPYoloELoss Loss refers to the sum of all the individual losses that usually are weighted with certain coefficients to weight their contributions.

Author Contributions: Funding: Conceptualization, Y.-C.C, H.-C.W. and A.M.; data curation, Y.-C.C and A.M.; formal analysis, C.-Y.K, R.K., S.S and A.M.; funding acquisition, Y.-C.C, A.M., and H.-C.W.; investigation, R.K. and A.M.; methodology, S.S, C.-K.C, H.-C.W., and A.M.; project administration, C.-K.C, A.M., and H.-C.W.; resources, C.-K.C, H.-C.W., and A.M.; software, C.-Y.K, T.-H.C, and A.M.; supervision, C.-Y.K, R.K., and H.-C.W.; validation, S.S and R.K.; writing—original draft, S.S, T.-H.C, R.K., and A.M.; writing—review and editing, R.K., A.M., and H.-C.W. All authors have read and agreed to the published version of the manuscript.

Institutional Review Board Statement: The study was conducted according to the guidelines of the Declaration of Helsinki and approved by the Institutional Review Board of the Institutional Review Board of Kaohsiung Armed Forces General Hospital (KAFGHIRB 112-018) and the Institutional Review Board of Dalin Tzu Chi General Hospital (B11301023).

Informed Consent Statement: Written informed consent was waived in this study because of the retrospective, anonymized nature of the study design.

Data Availability Statement: The data used in this study can be obtained from a reasonable request from the corresponding author (H.-C.W).

Conflicts of Interest: The authors declare no conflicts of interest.

References

1. Padilla, R., S.L. Netto, and E.A. Da Silva. *A survey on performance metrics for object-detection algorithms*. in *2020 international conference on systems, signals and image processing (IWSSIP)*. 2020. IEEE.
2. Justice, A.C., K.E. Covinsky, and J.A.J.A.o.i.m. Berlin, *Assessing the generalizability of prognostic information.* 1999. **130**(6): p. 515-524.
3. Hu, Y., Y. Liu, and Z. Liu. *A survey on convolutional neural network accelerators: GPU, FPGA and ASIC*. in *2022 14th International Conference on Computer Research and Development (ICCRD)*. 2022. IEEE.
4. Ganaie, M.A., et al., *Ensemble deep learning: A review.* 2022. **115**: p. 105151.

Disclaimer/Publisher's Note: The statements, opinions and data contained in all publications are solely those of the individual author(s) and contributor(s) and not of MDPI and/or the editor(s). MDPI and/or the editor(s) disclaim responsibility for any injury to people or property resulting from any ideas, methods, instructions or products referred to in the content.
